# Case Report: SCOUT MD™ four reflector technology—enhancing accuracy in breast lesion isolation

**DOI:** 10.3389/fsurg.2025.1683321

**Published:** 2025-10-08

**Authors:** Anna LaRussa, Sydney Caputo, Ali Sadeghi, Ralph Corsetti

**Affiliations:** 1 School of Medicine, Tulane University, New Orleans, LA, United States; 2 Cedars-Sinai Medical Center, Los Angeles, CA, United States; 3 University of British Columbia, Vancouver, BC, Canada; 4 SUNY Health Science Center, Brooklyn, NY, United States; 5 School of Medicine, Louisiana State University, New Orleans, LA, United States; 6 Sadeghi Center for Plastic Surgery, New Orleans, LA, United States; 7 School of Medicine, Boston University, Boston, MA, United States; 8 School of Medicine, Brown University, Providence, RI, United States; 9 Department of Surgery, Tulane University, New Orleans, LA, United States

**Keywords:** breast cancer, SCOUT, lesion resection, DCIS, reflector

## Abstract

We present a novel approach for localizing a large, non-palpable area of ductal carcinoma *in situ* (DCIS) for surgical resection using multiple SCOUT reflectors in a 45-year-old woman with DCIS of the left breast. Lesional resection using SCOUT MD™'s four unique reflectors was completed with real-time feedback from the SCOUT probe, providing information on distance, depth, and orientation around the perimeter of the calcifications. Successful DCIS resection was confirmed by post-resection tomosynthesis imaging of the partial mastectomy specimen, and negative margins were achieved. The patient synchronously chose contralateral breast reduction to achieve symmetry with the ipsilateral oncoplastic reduction mastoplasty. Advancements in SCOUT MD™ technology provide for improved intraoperative precision in the dissection with the use of four unique reflectors, while maintaining optimal cosmetic results by minimizing the removal of normal tissue. We believe these technological advancements in breast tumor localization will reduce rates of re-excision and the need for additional surgical management.

## Introduction

The original SCOUT™ technology, which employs radar detection of a single reflector, revolutionized the localization and excision of non-palpable breast lesions (1–7). With the use of SCOUT™, surgeons can perform resections with more efficiency compared with the traditional, cumbersome, and time-consuming method of wire localization (6–9). However, with just one reflector, lesion visualization is limited to the orientation and positioning of that reflector (10, 11). Furthermore, bracketing a lesion with multiple reflectors of identical structure often causes confusion as to which reflector signal and site is being detected. Advancements with SCOUT MD™ now provide four unique reflectors, which can be deployed around the lesion, allowing for more precise isolation and resection with real-time radar feedback from multiple locations at once (1, 2). The use of unique reflectors allows for differentiation between signals, orienting the surgeon to the precise depth and distance of the dissection.

In this report, we describe the use of novel SCOUT MD™ technology to localize a large area of non-palpable ductal carcinoma in situ (DCIS) for surgical resection using multiple, unique SCOUT MD™ reflectors. This technique provided improved accuracy in dissection and optimal cosmetic outcomes.

This case report describes a pre-menopausal G3P1 45-year-old woman with a medical history significant only for hypertension and negative for any personal history of malignancy. Her family history was significant for breast cancer in her maternal great grandmother, who was diagnosed at age 40, and in two of her maternal second cousins. Prior familial genetic testing had not been performed; however, her personal Myriad genetic testing was negative for any significant mutations, and management guidelines recommended routine screening. She gave birth at the age of 29, after which she did not breastfeed. She had a history of using estrogen-based birth control for 25 years.

**Figure 1:**
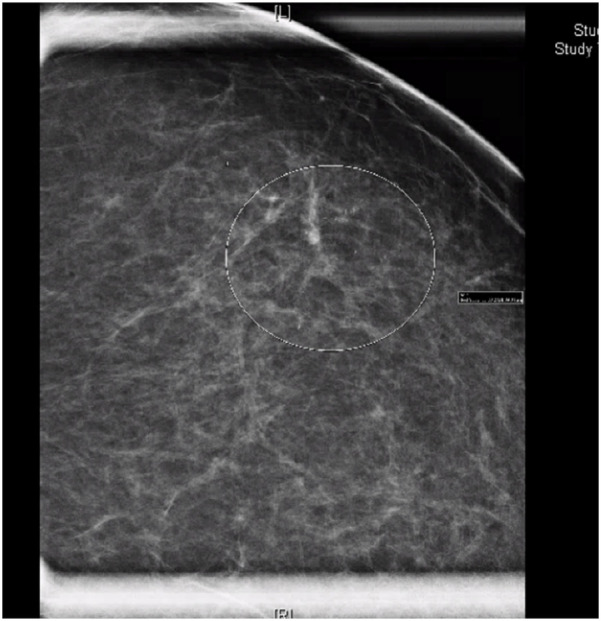
Mammogram of left breast demonstrating linear and segmental punctate calcifications over a 3.4 cm area, mid-depth. BI-RADS 4C.

The patient presented in 2024, after a routine mammogram screening identified indeterminate calcifications in the upper outer quadrant of the left breast, classified as Breast Imaging-Reporting and Data System (BI-RADS) 0. She denied any nipple discharge, breast discomfort, or other abnormalities on self-breast exams. Three days later, a subsequent diagnostic mammogram identified a BI-RADS 4C lesion with a 3.4 cm area of calcifications in a linear and segmental distribution, 7.9–11.3 cm from the nipple (Figure 1).

**Figure 2:**
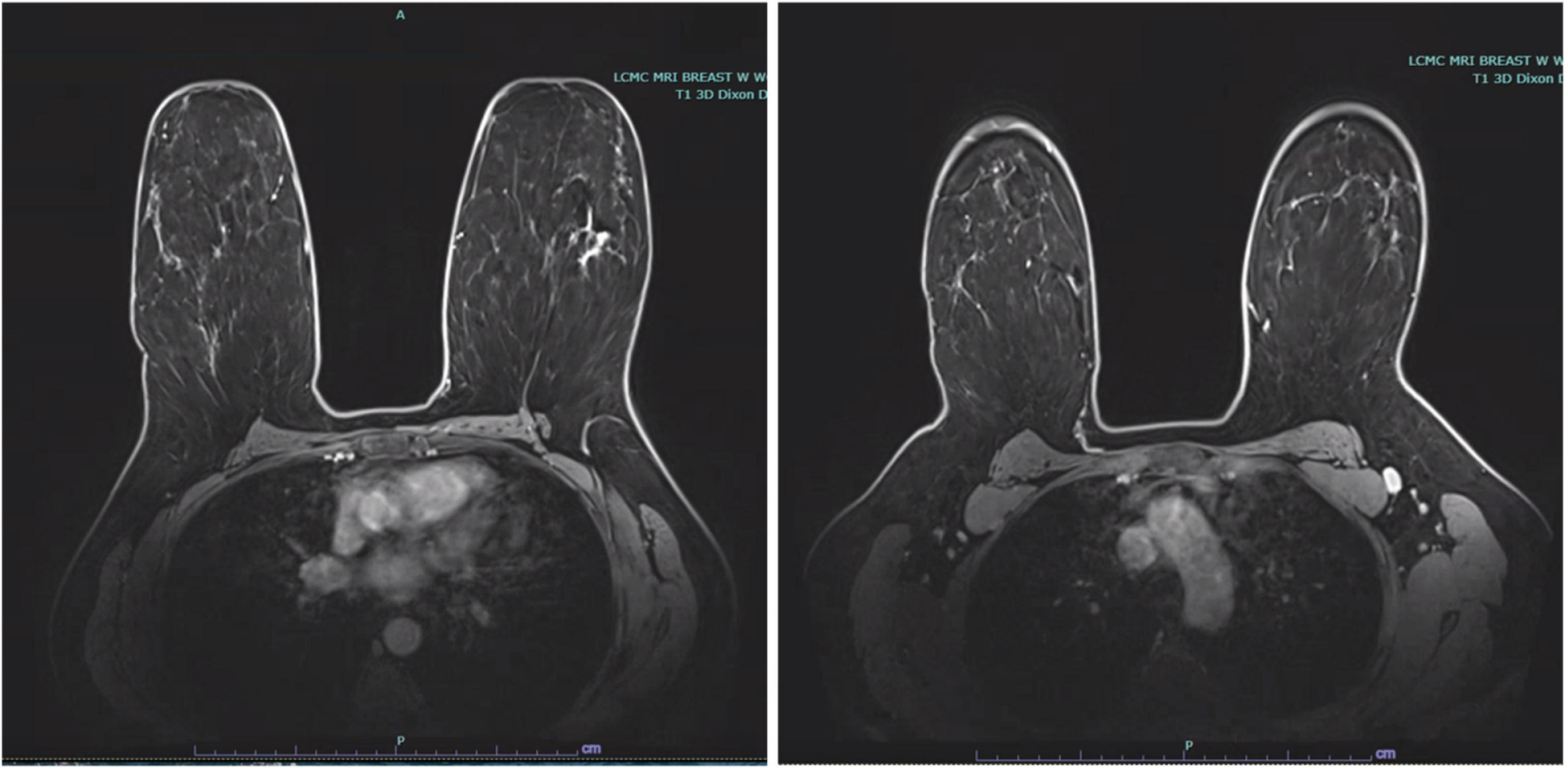
Bilateral breast MRI depicting malignancy in the upper outer quadrant of the left breast with non-mass enhancement measuring 5.2 cm, in addition to a prominent level 1 left axillary lymph node.

Stereotactic core needle biopsy completed 2 weeks later, confirmed high-grade DCIS with solid pattern, focal minimal comedonecrosis, and focal microcalcifications in the left breast. No lymphovascular invasion was identified. MRI correlated the non-palpable area of abnormality with a 5.2 cm area of non-mass enhancement. MRI also noted a prominent level 1 left axillary lymph node with no internal mammary lymphadenopathy (Figure 2).

The patient was counseled on mastectomy with or without reconstruction vs. breast conservation and radiation therapy. With G-sized breasts and the extent of the calcifications, the patient was a suitable candidate for bilateral breast reduction and mastopexy with left upper outer quadrant oncoplastic partial mastectomy (12–14).

Oncoplastic reduction techniques vary based on the location of the tumor to simultaneously preserve and shape the breast to enhance and achieve symmetry. This technique allows for larger resection volumes, ensuring that clear margins are achieved, and the remaining unresected breast tissue can be rearranged based on anatomic vascular breast flaps to obtain an esthetic improvement. This technique remains a plausible option in patients with large breast volumes seeking reconstruction with breast conservation therapy. To maximize obtaining optimal margins, a plan for SCOUT MD™ bracketing with four reflectors for localization and guided resection was made. A sentinel lymph node biopsy was also encouraged, given the extensive area of calcifications, the location of high-grade DCIS in the upper outer quadrant, and the anticipation for oncoplastic adjacent tissue transfer and rearrangement. With the 20% risk of upstaging in high grade DCIS, this would avoid the challenge of a difficult biopsy in the future. The patient was amenable to this technique, as her treatment goals focused on complete resection and cosmetic optimization.

## Methods

The patient described has provided consent for the publication of her case.

**Figure 3:**
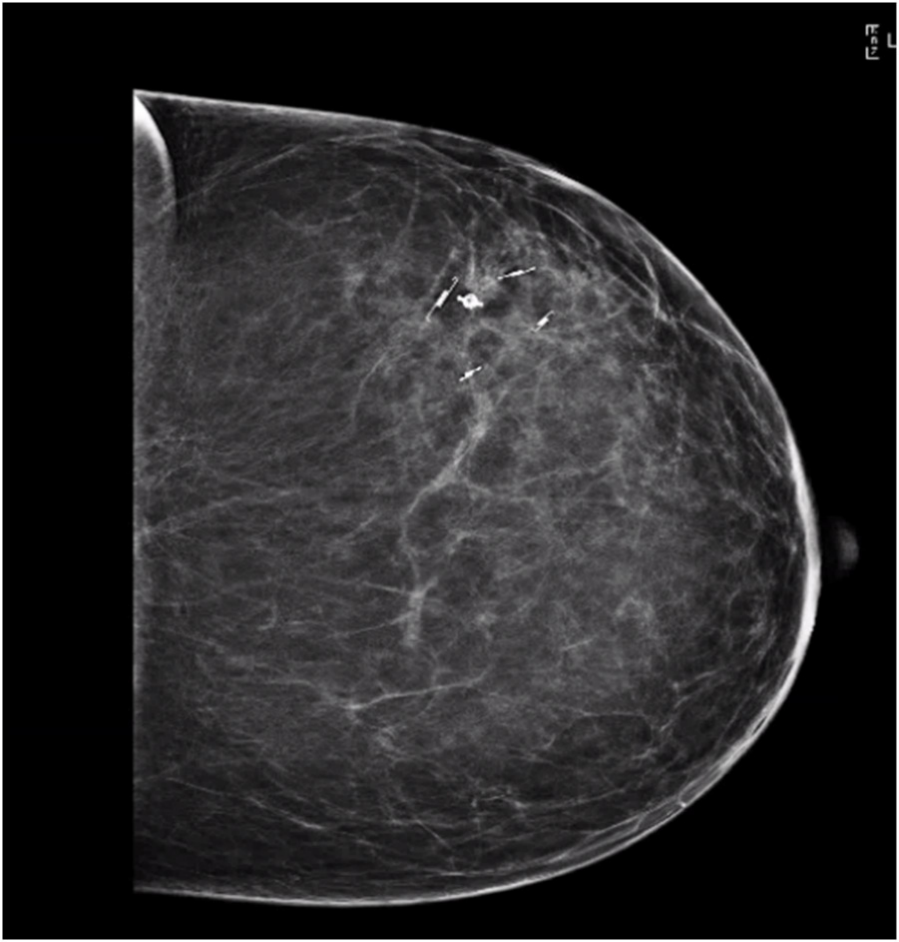
Ultrasound confirmation of SCOUT reflector locations around the calcified lesion. Calcifications surrounded by a double L shape proximally, double J shape laterally, double J shape medially, and JL shape distally.

Two days before the partial mastectomy, four unique SCOUT reflectors were each placed around the 4 cm area of calcifications. Specifically, a double L SCOUT was advanced into the proximal aspect of the group of calcifications. Similarly, two double J SCOUT reflectors were deployed in the lateral aspect and within the medical aspect of the calcifications. The breast was compressed in the lateral to medial positioning, and the J/L combination reflector was deployed in the distal aspect of the calcifications, posteriorly. Knowing we would take a full-thickness segment from the anterior skin edge to the posterior chest wall, these margins were not marked. Ultrasound confirmed the successful SCOUT reflector placements within the left breast (Figure 3).

On the day of surgery, lesional resection was completed with real-time feedback from the SCOUT probe, providing information on distance, depth, and orientation around the perimeter of the calcifications. The upper outer quadrant was dissected within the anterior subcutaneous plane, leaving a 1 cm thick skin flap in the upper outer quadrant. The two double J reflectors were identified medially and laterally using the SCOUT probe, and these were marked with a silk suture and single clips. Similarly, the anterior double L reflector was marked with two clips, and the posterior reflector (J/L) was marked with three clips. We dissected 2 cm grossly around all four SCOUT reflector signals. The dissection was advanced down to the pectoralis musculature of the chest wall. The anterior signal was dissected 5 cm from the nipple-areolar complex, and the distal location was dissected 15 cm from the nipple. Gross margins of >1 cm outside each reflector were provided using an intraoperative 3D specimen radiograph.

Given the extensive area of calcifications and high-grade DCIS in the upper outer quadrant as well as the complexity of the surgery, the sentinel lymph node biopsy was completed next. Through the superior lateral border of the pectoralis muscle, the clavipectoral fascia was opened to identify a deep level 1 left axillary sentinel lymph node, which was hot, blue, and palpable. The lymph node was dissected out and removed in its entirety.

**Figure 4:**
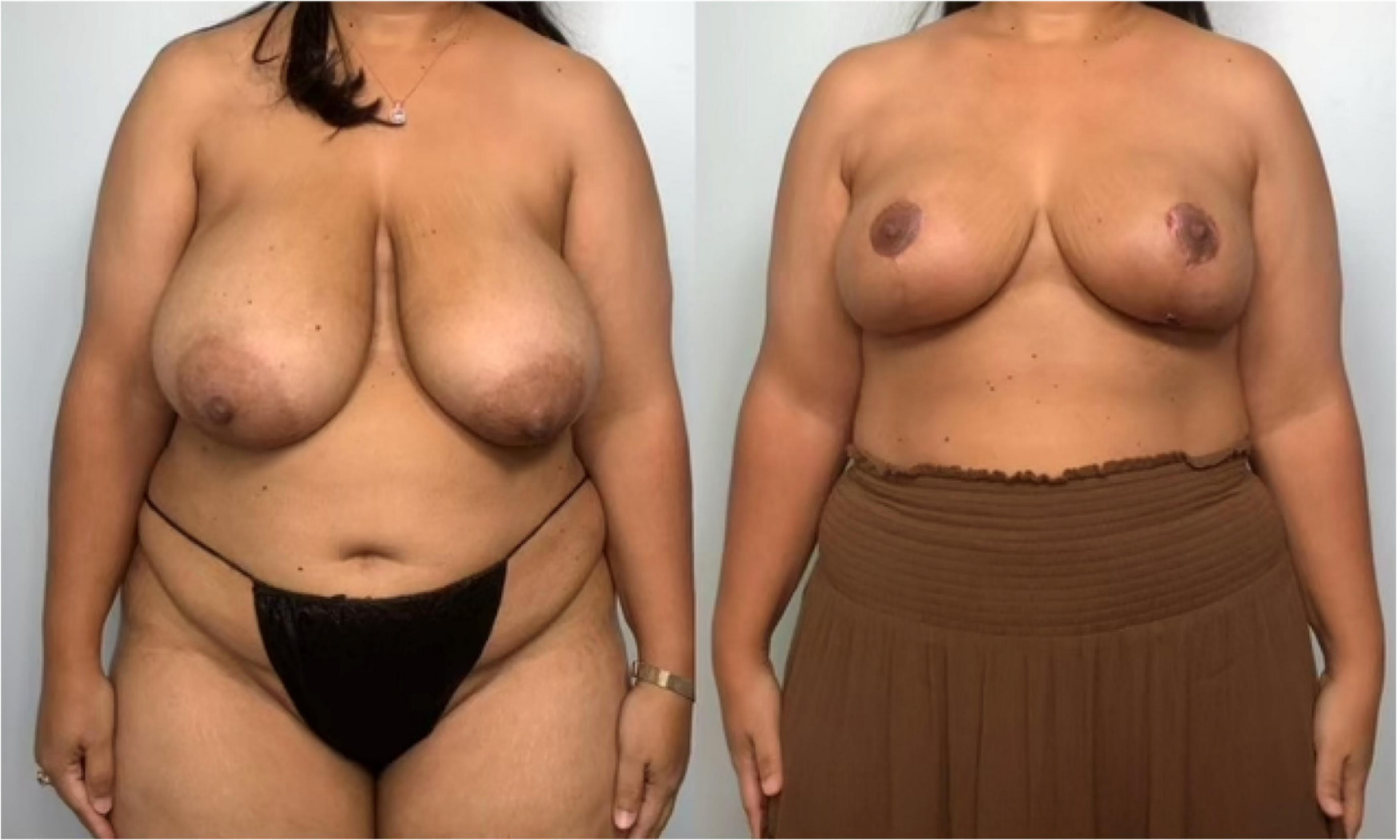
Patient before and after bilateral reduction mastoplasty. Initially, with G-sized breasts, she was reduced to C-sized breasts following the mastoplasty and lumpectomy.

Following resection, the plastic surgery team completed the bilateral breast reduction and lift, using anatomic vascular breast flaps to obtain esthetic improvement (Figure 4).

## Results

**Figure 5:**
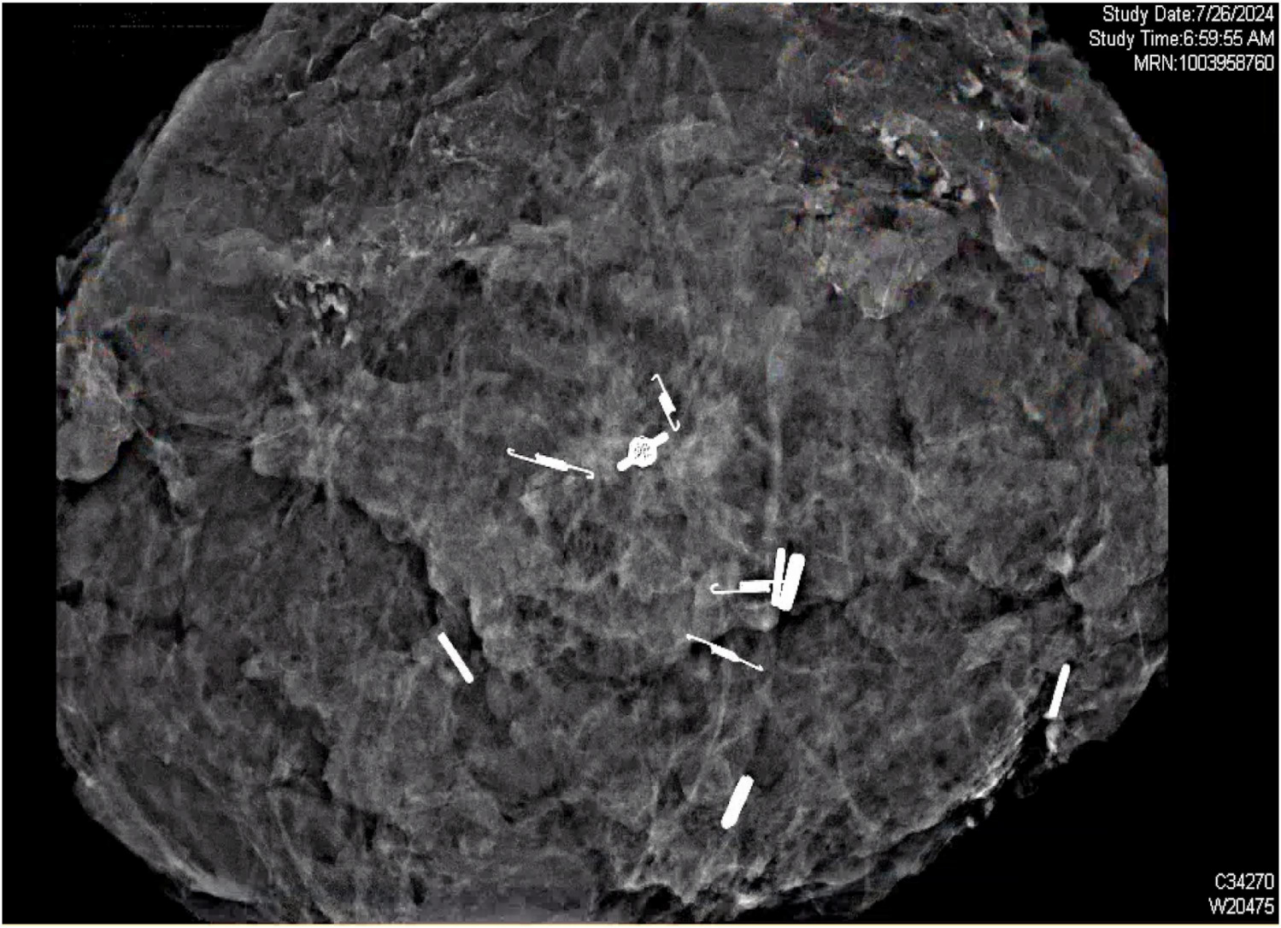
Post-resection ultrasound scan of the mass, identifying the four unique SCOUT reflectors surrounding the punctate calcifications in excised tissue.

Successful DCIS resection with negative margins on pathology was achieved using SCOUT MD™ localization. With guided feedback from the four unique SCOUT signals, the borders of the non-palpable lesion were clearly identified, and an 18.5 cm × 15.7 cm × 8.5 cm specimen, weighing 430.5 g, was resected from the left breast. A single left axillary sentinel lymph node, identified by radioactive tracer, was removed, ultimately testing negative for any evidence of carcinoma on frozen section. The contralateral right breast reduction and lift was completed by the plastic surgery team, with 900 g of tissue from the benign reduction. Intraoperative tomosynthesis specimen radiograph and imaging confirmed all margins were grossly clear by at least 2 cm around the calcifications. Post-resection imaging of the left breast specimen confirmed the target SCOUT markers at the expected locations proximally, laterally, medially, and distally around the punctate calcifications (Figure 5).

Final histopathology of the non-palpable lesion confirmed at least 2 mm of high-grade DCIS, solid and cribriform type with comedonecrosis. Negative margins were confirmed with the closest margin being the anterior margin at 5.5 mm. Immunohistochemical staining was positive for ADH4, P63, CK5/14, and CK7/19+.

The patient's Memorial Sloan Kettering Cancer Center risk of local recurrence was 11% and 18% at 5 and 10 years, respectively, which significantly reduced to 4% and 7% with the addition of radiation therapy (15). Given this information, she chose to receive adjuvant radiotherapy to the volume-reduced left breast for 6.5 weeks. She recovered from surgery well and tolerated radiotherapy with no difficulties. Today, she remains disease-free and satisfied with her symmetric oncoplastic partial mastectomy and breast reduction. She follows up every 6 months in the clinic for continued clinical surveillance and high-risk screening.

## Discussion

The original SCOUT technology offers ease and precision to breast lesion isolation by utilizing radar detection of a small reflector placed in the tissue. However, challenges to the original SCOUT technology include a limited multidimensional perspective of the lesion, given the use of only one reflector signal. Strategies to bracket an impalpable lesion with multiple identical reflectors provides for more detailed orientation of the lesion; however, the reflector signals are easily confused, given poor differentiation between signal feedback.

Advancements with SCOUT MD™ now provide four unique reflectors, which can be deployed around the lesion, allowing for more precise isolation and resection. With real-time radar feedback from the unique reflectors surrounding the lesion, surgeons can differentiate between the signals and are more precisely oriented to the depth and distance of the dissection, optimizing margins of resection.

The use of SCOUT MD™ in tumor localization offers further precision of tumor resection, while maintaining patient and facility flexibility for scheduling (2–5). These advancements in SCOUT MD™ technology, employing four unique reflectors, provide precise information on orientation and depth of lesional borders throughout the resection. Utilizing four reflectors provides a full, three-dimensional orientation of the impalpable lesion during the dissection, a marked improvement from the two-dimensional orientation provided by only two reflectors. Furthermore, SCOUT MD™ facilitates optimal cosmetic results by minimizing the removal of normal tissue.

When compared with different wire-free techniques for preoperative localization of non-palpable breast lesions, SCOUT MD™ has certain advantages that promote patient satisfaction and surgical precision. Compared to Radioactive Seed Localization (RSL), SCOUT MD™ is non-radioactive, eliminating concerns for radiation safety and the need for nuclear medicine-approved facilities (10). While our case warranted a sentinel lymph node biopsy, this advantage still provided ease during reflector placement and DCIS resection. Furthermore, when compared to Magseed technology, SCOUT MD™ offers four unique signals to promote clarity of orientation during the dissection, and the localization is not limited by reflector depth (16).

We believe these technological advancements in breast tumor localization will reduce rates of re-excision and the need for additional surgical management.

## Data Availability

The original contributions presented in the study are included in the article/Supplementary Material; further inquiries can be directed to the corresponding author.
